# The effects of in-hospital deprescribing on potential prescribing omission in hospitalized elderly patients with polypharmacy

**DOI:** 10.1038/s41598-021-88362-w

**Published:** 2021-04-26

**Authors:** Miho Kaminaga, Junpei Komagamine, Shinpei Tatsumi

**Affiliations:** 1 Department of Pharmacy, National Hospital Organization Tochigi Medical Center, 1-10-37, Nakatomatsuri, Utsunomiya, Tochigi Japan; 2 Department of Internal Medicine, National Hospital Organization Tochigi Medical Center, 1-10-37, Nakatomatsuri, Utsunomiya, Tochigi Japan

**Keywords:** Health care, Geriatrics

## Abstract

No studies to investigate the effect of a deprescribing intervention on the occurrence of potential prescribing omissions (PPOs) among elderly patients with polypharmacy have been conducted. Therefore, the effect of deprescribing on PPOs among elderly patients with polypharmacy was investigated. All 121 consecutive elderly patients who received in-hospital deprescribing interventions were evaluated. The primary outcome was any occurrence of PPOs based on the 2015 STOPP/START criteria. The proportion of patients who had any PPOs significantly increased after the deprescribing interventions (52.9% vs 77.7%, *p* < 0.001). In the multivariable analysis, older age was the only independent risk factor associated with an increased risk of any PPOs after the deprescribing interventions (OR 1.08, 95% CI 1.01 to 1.16). In-hospital deprescribing interventions for elderly patients with polypharmacy may increase the occurrence of PPOs. Further study is warranted to investigate the effects on clinical outcomes of the increased occurrence of PPOs due to the deprescribing intervention.

## Introduction

Polypharmacy refers to the use of multiple medications. In various studies, polypharmacy has been varyingly defined. Some investigators have defined it as the use of unnecessary medications^1^, while others have defined it as the use of two or more medications for the same conditions^2^. However, the most commonly used definition of polypharmacy is the numerical definition of polypharmacy of regular use of five or more medications regardless of whether they are necessary or unnecessary^3^, although there is no universal consensus for the optimal number of concomitant medications that would be defined as polypharmacy^3,4^.

Polypharmacy is common among elderly patients because they have multiple comorbidities that lead to the use of multiple medications^5^. Population-level data reports that 30 to 40% of noninstitutional people aged more or 65 years old take 5 or more medications^6,7^. The prevalence of polypharmacy roses to up to 60% in long-term care facilities^8^ and in-hospital settings^9,10^. Although prescribing medications is critical for elderly patient care, as the number of medications increases, the risk of adverse drug reactions increases^11^. In fact, polypharmacy for elderly patients is associated with an increased risk of adverse drug reactions^12,13^, fall injuries^14^, frailty^15^, and mortality^16,17^. Moreover, polypharmacy is also associated with nonadherence to medications^18^ and inappropriate prescription^19,20^. Therefore, some strategies to improve polypharmacy and inappropriate prescription among elderly patients are needed.

One strategy to resolve these problems is deprescribing, which is the systematic process of identifying and discontinuing medications for which the potential harms outweigh the potential benefits within the context of an individual patient’s care goals as supervised by healthcare professionals^21^. Deprescribing interventions can reduce inappropriate medications and medication-related problems^22–24^ and improve adherence to medications^25^. However, recent meta-analyses reported that deprescribing interventions cannot reduce the risk of hospital admission and death^22–24^. Thus, deprescribing interventions are still not proven to have a beneficial effect on clinically important outcomes except medication-related adverse events. It remains uncertain why deprescribing interventions cannot improve these outcomes.

Underprescribing is another important aspect of an inadequate prescription practice. The omission of drug therapy indicated for the prevention or treatment of specific diseases or conditions is associated with adverse outcomes^26^. Therefore, geriatric experts have recommended reducing potential prescribing omissions (PPOs) as much as possible among elderly patients^27^. However, the prevalence of PPOs is high in elderly patients^20^. Moreover, patients who take more medications have more PPOs^28,29^. Although the true reason for this remains unknown, it is thought that physicians may be reluctant to add new medications to polypharmacy^29^. In addition, the limited life expectancy and frailty of elderly patients may prevent physicians from prescribing preventive medications.

The relationship between deprescribing and underprescribing is complicated. For example, statins are beneficial for the secondary prevention of cardiovascular disease (CVD), even in elderly patients. Therefore, unless statins have been prescribed for elderly patients with CVD, this would be considered an inappropriate prescribing omission. However, statin use may be discouraged for the secondary prevention of CVD in patients with advanced illness or limited life expectancy^30^. In this case, deprescribing statins for these patients would be justified. However, given that estimation of the prognosis of elderly patients is difficult^31^, physicians’ underestimation of patients’ life expectancy may result in an unintentional increase in PPOs due to deprescribing. Moreover, an intervention to improve PPOs is not included in the deprescribing intervention. Therefore, a lack of efficacy of the deprescribing intervention on clinically important outcomes may be caused by the increase or lack of change of PPOs.

Nonetheless, no studies have been conducted to investigate the effect of the deprescribing intervention on the occurrence of PPOs among elderly patients with polypharmacy. Thus, our aim was to investigate the effect of the deprescribing intervention on the occurrence of PPOs among elderly patients with polypharmacy. The risk factors associated with the occurrence of PPOs after the in-hospital deprescribing interventions were also determined.

## Results

A total of 121 elderly patients were included. The mean age of the patients was 80.5 (SD 7.4) years, 83 (68.6%) were women, and the mean Charlson Comorbidity Index score was 1.9 (SD 1.6) (Table 1 and Table S1). Twenty-four patients (19.8%) had dementia, 15 (12.4%) were nursing home residents, and 77 (63.6%) were able to walk independently. The most common admission ward was the orthopedic ward (n = 86, 85.2%), followed by the general surgery ward (n = 7, 5.8%). Only one patient (0.9%) died during the index hospitalization.

**Table 1:** Characteristics of 121 hospitalized elderly patients who received the in-hospital deprescribing intervention.

Characteristics^a^	Total	Presence of PPOs after the deprescribing intervention	
		Yes (n = 94)	No (n = 27)
Mean age, year (SD)	80.5 (7.4)	81.4 (7.2)	77.1 (7.3)
Woman	83 (68.6)	66 (70.2)	17 (63.0)
**Location before the index admission**			
Home	104 (86.0)	79 (84.0)	25 (92.6)
Nursing home	15 (12.4)	14 (14.9)	1 (3.7)
Other hospitals	2 (1.7)	1 (1.1)	1 (3.7)
**Ambulatory status before admission**			
Independence	77 (63.6)	56 (59.6)	21 (77.8)
Partial or totally dependence	44 (36.4)	38 (40.4)	6 (22.2)
Mean Charlson Comorbidity Index score (SD)	1.9 (1.6)	2.0 (1.6)	1.5 (1.6)
**Past medical history**			
Hypertension	97 (80.2)	72 (76.6)	25 (92.6)
Dyslipidemia	43 (35.5)	30 (31.9)	13 (48.2)
Diabetes mellitus	25 (20.7)	16 (17.0)	9 (33.3)
Asthma or COPD	5 (4.1)	3 (3.2)	2 (7.4)
Dementia	24 (19.8)	22 (23.4)	2 (7.4)
Ischemic stroke	18 (14.9)	15 (16.0)	3 (11.1)
Ischemic heart disease	10 (8.3)	7 (7.5)	3 (11.1)
Chronic kidney disease	8 (6.6)	7 (7.5)	1 (3.7)
Heart failure	5 (4.1)	5 (5.3)	0 (0.0)
Atrial fibrillation	18 (14.9)	14 (14.9)	4 (14.8)
**Admission ward**			
Orthopedic surgery	86 (85.2)	83 (88.3)	18 (66.7)
General surgery	7 (5.8)	5 (5.3)	2 (7.4)
Others^b^	13 (10.7)	6 (6.4)	7 (25.9)
In-hospital death	1 (0.8)	1 (1.1)	0 (0.0)

*COPD* chronic obstructive pulmonary disease, *PPO* potential prescribing omission, *SD* standard deviation.

^a^Values are expressed as the number with the percentage of the total number, unless otherwise stated.

^b^Include neurosurgery (n = 5), oral surgery (n = 4), urology (n = 3), dermatology (n = 3).

Although a mean of 0.8 medications were newly started during the index hospitalization, the mean number of total medications was significantly reduced after the deprescribing interventions (9.1 medications vs 4.7 medications, *p* < 0.001) (Table 2, Table S2, and Supplementary Data). The proportion of patients who took any PIMs was also significantly reduced after the deprescribing interventions (71.1% vs 43.0%, *p* < 0.001). However, the proportion of patients who had any PPOs significantly increased after the deprescribing interventions (52.9% vs 77.7%, *p* < 0.001). The most common type of PPO identified after the deprescribing interventions was musculoskeletal system drugs. More than half of all patients had at least one PPO for the musculoskeletal system. In the multivariable analysis, older age was the only independent risk factor associated with an increased risk of any PPOs after the deprescribing interventions (OR 1.08, 95% CI 1.01 to 1.16) (Table 3).

**Table 2:** Change in the numbers of all medications, PIMs, and PPOs after the in-hospital deprescribing intervention.

Characteristics^a^	In-hospital deprescribing		*P* value^b^
	Before (at admission)	After (at discharge)	
**Number of total medications**^**b**^			
Mean (SD)	9.1 (2.6)	4.7 (2.5)	< 0.001
Five or more medications	119 (98.4)	61 (50.4)	< 0.001
**Potentially inappropriate medications**^**b,c**^			
Any use	86 (71.1)	52 (43.0)	< 0.001
Mean (SD)	1.2 (1.1)	0.6 (0.8)	< 0.001
**Potential prescribing omissions**^**b,c**^			
Any occurrence	64 (52.9)	94 (77.7)	< 0.001
Mean number (SD)	0.5 (0.5)	2.1 (1.6)	< 0.001

*COPD* chronic obstructive pulmonary disease, *PIM* potentially inappropriate medication, *PPO* potential prescribing omission, *SD* standard deviation.

^a^Values are expressed as the number with the percentage of the total number, unless otherwise stated.

^b^Comparisons between the time at admission and discharge were performed by using Fisher’s exact test and Student’s t-test for categorical and continuous variables, respectively. The level of statistical significance was set at 5%.

^c^Based on the 2015 STOPP/START criteria.

**Table 3:** Results of multivariable analysis for factors associated with the presence of PPOs at discharge.

Variables	Odds ratios (95% CI)	*P* value
Older age	1.08 (1.01–1.16)	0.02
Female sex	1.65 (0.63–4.36)	0.31
Residence at nursing home	2.37 (0.25–22.54)	0.45
Independent walking	0.64 (0.20–2.06)	0.45
Number of medications at discharge	1.09 (0.90–1.33)	0.37
Charlson Comorbidity Index score	1.12 (0.81–1.55)	0.49

The following variables were used: age, sex, residence, Charlson Comorbidity Index score, ambulatory status, and number of medications at discharge. The level of statistical significance was set at 5%.

*CI* confidence interval, *PPO* potential prescribing omission.

## Discussion

This is the first study to investigate the effect of a deprescribing intervention on the occurrence of PPOs among elderly patients with polypharmacy. Our study revealed that the in-hospital deprescribing intervention increased the occurrence of PPOs, although it significantly reduced the total number of medications and the use of any PIMs. Older age was the only independent predictive factor for any use of PPOs after the deprescribing intervention.

Several explanations for the increase in the use of PPOs after the in-hospital deprescribing intervention can be considered. First, some potentially necessary medications based on the 2015 START criteria might be judged to be unnecessary during the deprescribing intervention after sufficient assessment of these medications by the physicians who performed the deprescribing intervention. A previous study reported that one of the independent risk factors for not following the START criteria was patients’ inability to walk^32^. Moreover, given that older age was an independent predictive factor for the occurrence of PPOs after the deprescribing intervention, physicians might think that deprescribing potentially necessary medications based on the START criteria outweighs starting or continuing these medications from the viewpoint of the severe disability and limited life expectancy of the patients. If so, the occurrence of PPOs after the deprescribing intervention may be safe.

Second, the present study included only patients or their caregivers who chose to participate in the deprescribing intervention. Therefore, there might be some preferences on the part of the patients or their caregivers for the deprescribing intervention rather than starting new medications. Third, not starting or continuing potentially necessary medications may be caused by newly published evidence available after the release of the 2015 STOPP/START criteria^32^ and the limitations of past evidence. For example, a recent randomized controlled trial suggested that there was a lack of benefit of bisphosphonate with regard to preventing fractures in frail elderly women with osteoporosis^33^. Moreover, a past randomized controlled trial showing the efficacy of bisphosphonate excluded patients who could not walk independently before hip fracture^34^. This evidence may lead physicians not to start or continue potentially necessary medications among frail older patients.

Given that the effect of the deprescribing intervention on improving clinically important outcomes remains uncertain, further study is warranted to confirm our findings at other hospitals and investigate whether the increase in the occurrence of PPOs after the deprescribing intervention is safe or harmful for elderly patients with polypharmacy.

Several limitations need to be mentioned. First, this study had no control group that did not undergo the in-hospital deprescribing intervention. Therefore, the changes in PIMs and PPOs after the deprescribing interventions in this study did not necessarily derive from the effects of the intervention. However, given that the number of total medications and PIMs were unchanged or increased without the deprescribing intervention^35,36^, our results probably reflect the effect of the deprescribing intervention. Second, the information that was needed to evaluate PPOs was collected retrospectively. Therefore, the assessment of the occurrence of PPOs might be inaccurate^37^. Third, a single-center study design limits the generalization of our results. Fourth, only 121 elderly patients received the deprescribing intervention, although the interventions were performed over a 3-year period. Therefore, the low rate of recruitment among the patients who met the screening criteria for the deprescribing intervention also limits the generalizability of the present study. Fifth, geriatricians were not involved in the deprescribing intervention, although most past studies regarding deprescribing intervention also involved no geriatricians^22,25^. Sixth, our method of the deprescribing intervention may be implicit. Therefore, its external validity may be limited. However, we used a similar approach as the past study did^38^. Moreover, the method of deprescribing interventions used in some past studies is also implicit^22^. Finally, we did not collect information on medications after discharge. Given that discrepancies between medications at discharge and after discharge are common^39^, it is uncertain whether the effect of the deprescribing intervention continued after discharge.

## Methods

### Study setting and design

A retrospective single-center observational study was conducted by using medical electronic records to investigate the effect of deprescribing interventions on PPOs among hospitalized elderly patients with polypharmacy. Our hospital is a 350-bed acute care hospital, and it is one of the largest community hospitals in Utsunomiya, which has a population of approximately 0.5 million and is located in the central part of Japan. Geriatricians and geriatric care units are not available in our hospital. The study was approved by the Medical Ethics Committee of the National Hospital Organization Tochigi Medical Center (No. 30-4). This study was conducted in accordance with the Ethical Guidelines for Epidemiological Research in Japan and was conducted in accordance with the Declaration of Helsinki. The need for individual informed consent was formally waived by the Medical Ethical Committee of the National Hospital Organization Tochigi Medical Center because deidentified data were collected without contacting the patients. However, as per Japanese Ethical Guidelines, we displayed an opt-out statement in the waiting room and webpage of the hospital to inform the study and provide the opportunity to refuse the use of data for the patients.

### In-hospital deprescribing intervention

Our hospital started performing in-hospital deprescribing interventions for hospitalized elderly patients with polypharmacy in the orthopedic ward in January 2015^40^. Other surgical wards, such as general surgery and urology, were added as target wards. The prevalence of polypharmacy is high among elderly patients hospitalized due to fractures^41^. Moreover, in our hospital, deprescribing intervention has been a routine care for elderly patients in the internal medicine ward since 2014^42^. Therefore, we chose the orthopedic ward rather than the internal medicine ward. Patients aged 65 years old or older who were hospitalized in a target ward and took 5 or more regular medications were screened and contacted by pharmacists after admission. In addition, a list of medications that were judged to be potentially inappropriate medication (PIM) based on the 2015 screening tool of older persons’ prescriptions (STOPP) criteria^27^ were documented at medical records by the pharmacists. Then, patients consulted with internal medicine physicians who deprescribed inappropriate or unnecessary medications, if possible, during the index hospitalization. The internal medicine physicians evaluated the appropriateness of the polypharmacy and changed medications as needed. The appropriateness of medications was determined based on the STOPP criteria and the following^38^: (1) Does evidence exist supporting the use of the medication for the indication given for this patient? (2) Does an indication seem valid and relevant given this patient’s age and disability level? (3) Do the known possible adverse reactions of the medication outweigh the possible benefits for this patient? (4) Has any adverse event that may be related to the medication occurred? (5) Is there alternative medication or nonpharmacological treatment that may be safer and similarly effective compared with the current medication? The deprescribing intervention was performed until discharge. Four internal medicine physicians and three clinical pharmacists were mainly involved in this process. One of those physicians was trained in geriatrics, and all physicians received two lectures about polypharmacy and deprescribing annually since 2012. All three pharmacists, including MK and ST, were trained in geriatric pharmacy.

### Inclusion and exclusion criteria

All consecutive elderly patients who received the in-hospital deprescribing intervention from internal medicine physicians from January 2015 to December 2017 were included. Patients aged less than 65 years old were excluded. The target patients were identified in the database of our hospital. During the study period, 123 patients received the in-hospital deprescribing intervention from internal medicine physicians. After excluding two patients aged less than 65 years old, a total of 121 patients were included in the final analysis.

### Data collection and outcome measures

Pharmacists (MK, ST) reviewed the electronic medical records and retrieved information on patient age, sex, past medical history, medication use, Charlson Comorbidity Index score^43^, renal function, and prognosis. The Charlson Comorbidity Index score was determined via chart review. Information on medications at admission was based on a comprehensive list of current medications that was compiled by pharmacists after admission. Information on medications at discharge was based on the discharge prescriptions issued by physicians. For renal function, the best value during the index hospitalization was adopted.

The primary outcome was the proportion of patients who had any PPOs. The secondary outcome was the proportion of patients who took any potentially inappropriate medications (PIMs). We defined PPOs and PIMs based on the 2015 screening tool of older persons’ prescriptions (STOPP) and the screening tool to alert to right treatment (START) criteria^27^. Section I of the START criteria (vaccine criteria) was excluded because information on vaccination was often not documented in the medical electronic records in our hospital. The total number of medications was also evaluated. Oral medications, inhalers, and injections were included. However, eye drops, intranasal infusers, topical medications, and over-the-counter drugs were excluded. As-needed medications were also excluded. Changes in the primary and secondary outcomes before and after the deprescribing intervention were investigated. The data collection and assessment of outcomes were performed from October 2019 to December 2019.

### Statistical analyses

Baseline characteristics of patients are described with descriptive statistics. For the changes in the primary outcome, a comparison of the proportions of patients who had any PPOs at admission and at discharge was performed by using Fisher’s exact test. The same analysis was performed for the proportions of patients who took any PIMs. For the total number of medications, Student’s t-test was used to compare the totals at admission and at discharge. To determine the predictive factors associated with the occurrence of PPOs at discharge, a multivariable analysis using binary logistic regression was performed to examine the associations between PPOs at discharge and the following variables: age, sex, residence before admission, Charlson Comorbidity Index, ambulatory status, and number of medications at discharge. Stata V.15 (LightStone, Tokyo Japan) was used for these analyses. The level of statistical significance was set at 5%.

## Conclusions

The in-hospital deprescribing intervention increased the occurrence of PPOs. Older age was the only independent predictive factor associated with the occurrence of PPOs after the deprescribing intervention. Further study is warranted to investigate the effect of the increased occurrence of PPOs due to the deprescribing intervention on clinical outcomes.

## Supplementary Information

Supplementary Information 1.

Supplementary Information 2.
