# Supplementary material for: The effects of in-hospital deprescribing on potential prescribing omission in hospitalized elderly patients with polypharmacy
Source: Sci Rep. 2021 Apr 26;11:8898. doi: 10.1038/s41598-021-88362-w (PMC8076214; doi:10.1038/s41598-021-88362-w)
Supplement: Supplementary file 1 — Supplementary Information 1. [file 41598_2021_88362_MOESM1_ESM.pdf]

The effects of in-hospital deprescribing on potential prescribing omission in hospitalized elderly patients with polypharmacy.

Authors:

Miho Kaminaga

Department of Pharmacy, National Hospital Organization Tochigi Medical Center, 1-10-37, Nakatomatsuri, Utsunomiya, Tochigi, Japan.

Junpei Komagamine

Department of Internal Medicine, National Hospital Organization Tochigi Medical Center, 1-10-37, Nakatomatsuri, Utsunomiya, Tochigi, Japan.

Shinpei Tatsumi

Department of Pharmacy, National Hospital Organization Tochigi Medical Center, 1-10-37, Nakatomatsuri, Utsunomiya, Tochigi, Japan.

Table S1. Characteristics of 121 hospitalised elderly patients who received the in-hospital deprescribing intervention.

Characteristics[a]	Total	Presence of PPOs after the deprescribing intervention	
		Yes (n = 94)	No (n = 27)
Age			
Median (IQR)	82 (75 – 85)	82 (77 – 87)	77 (71 – 82)
Mean (SD)	80.5 (7.4)	81.4 (7.2)	77.1 (7.3)
Woman	83 (68.6)	66 (70.2)	17 (63.0)
Location before the index admission			
Home	104 (86.0)	79 (84.0)	25 (92.6)
Nursing home	15 (12.4)	14 (14.9)	1 (3.7)
Other hospitals	2 (1.7)	1 (1.1)	1 (3.7)
Ambulatory status before admission			
Independence	77 (63.6)	56 (59.6)	21 (77.8)
Partial dependence	22 (18.2)	19 (20.2)	3 (11.1)
Dependence	22 (18.2)	19 (20.2)	3 (11.1)
Current smoker	24 (19.8)	23 (24.5)	1 (3.7)
Regular drinker	19 (15.7)	14 (14.9)	5 (18.5)
Charlson Comorbidity Index score			
Median (IQR)	2 (1 – 3)	2 (1 – 3)	1 (1 – 2)
Mean (SD)	1.9 (1.6)	2.0 (1.6)	1.5 (1.6)
Past medical history			
Hypertension	97 (80.2)	72 (76.6)	25 (92.6)
Dyslipidemia	43 (35.5)	30 (31.9)	13 (48.2)
Diabetes mellitus	25 (20.7)	16 (17.0)	9 (33.3)
Asthma or COPD	5 (4.1)	3 (3.2)	2 (7.4)
Dementia	24 (19.8)	22 (23.4)	2 (7.4)
Haemorrhagic stroke	12 (9.9)	10 (10.6)	2 (7.4)
Ischaemic stroke	18 (14.9)	15 (16.0)	3 (11.1)
Ischaemic heart disease	10 (8.3)	7 (7.5)	3 (11.1)
Chronic kidney disease	8 (6.6)	7 (7.5)	1 (3.7)
Heart failure	5 (4.1)	5 (5.3)	0 (0.0)
Atrial fibrillation	18 (14.9)	14 (14.9)	4 (14.8)
Osteoporosis	28 (23.1)	27 (28.7)	1 (3.7)
Fracture	45 (37.2)	41 (43.6)	4 (14.8)

Rheumatic disease	11 (9.1)	11 (11.7)	0 (0.0)
Depressive disorder	8 (6.6)	7 (7.5)	1 (3.7)
Active cancer	10 (8.3)	5 (5.3)	5 (18.5)
Admission ward			
Orthopaedic surgery	86 (85.2)	83 (88.3)	18 (66.7)
General surgery	7 (5.8)	5 (5.3)	2 (7.4)
Others[b]	13 (10.7)	6 (6.4)	7 (25.9)
Estimated GFR[c], ml/min/1.73m^2			
Median (IQR)	65 (48 – 80)	65 (47 – 79)	65 (53 – 82)
Mean (SD)	65 (22)	64 (22)	67 (22)
Less than 60 ml/min/1.73m^2	50 (41.3)	40 (42.6)	10 (37.0)
Number of medications at admission			
Median (IQR)	9 (7 – 11)	9 (8 – 11)	8 (7 – 10)
Mean (SD)	9.1 (2.6)	9.3 (2.6)	8.4 (2.6)
Five or more medications	119 (98.4)	92 (97.9)	27 (100.0)
Number of newly started medications during the index hospitalisation			
Median (IQR)	0 (0 – 2)	0 (0 – 1)	1 (0 – 2)
Mean (SD)	0.8 (1.2)	0.8 (1.2)	1.0 (1.2)
Number of medications at discharge			
Median (IQR)	5 (3 – 6)	4 (3 – 6)	5 (3 – 6)
Mean (SD)	4.7 (2.5)	4.7 (2.6)	4.7 (2.2)
Five or more medications	61 (50.4)	46 (48.9)	15 (55.6)
In-hospital death	1 (0.8)	1 (1.1)	0 (0.0)

[a]Values are expressed as the number with the percentage of the total number, unless otherwise stated.

[b]Include Neurosurgery (n = 5), Oral Surgery (n = 4), Urology (n = 3), Dermatology (n = 3).

[c]Based on the modification of diet in renal disease equation.

COPD: chronic obstructive pulmonary disease; GFR: glomerular filtration rate; IQR: interquartile range; PPO: potential prescribing omission; SD: standard deviation.

Table S2. Change in the numbers of all medications, PIMs, and PPOs after the in-hospital deprescribing intervention.

**Characteristics**[a]	**In-hospital deprescribing**		***P*-value**[b]
	**Before (At admission)**	**After (At discharge)**	
Number of total medications[b]			
Mean (SD)	9.1 (2.6)	4.7 (2.5)	< 0.001
Five or more medications	119 (98.4)	61 (50.4)	< 0.001
Potentially inappropriate medications[b,c]			
Any use	86 (71.1)	52 (43.0)	< 0.001
Mean (SD)	1.2 (1.1)	0.6 (0.8)	< 0.001
STOPP criteria			
Section A: Drug indication			
Any drug prescribed without an evidence-based clinical indication (A1)	4 (3.3)	1 (0.8)	
Any duplicate drug class prescription e.g. two concurrent NSAIDs, SSRIs, loop diuretics, ACE inhibitors, anticoagulants (A3)	6 (5.0)	4 (3.3)	
Section B: Cardiovascular system			
Loop diuretic as first-line treatment for hypertension (B6)	6 (5.0)	0 (0.0)	
Coagulation system			
Aspirin plus clopidogrel as secondary stroke prevention, unless the patient has a coronary stent(s) inserted in the previous 12 months or concurrent acute coronary syndrome or has a high grade symptomatic carotid arterial stenosis (C4)	1 (0.8)	1 (0.8)	
Antiplatelet agents with vitamin K antagonist, direct thrombin inhibitor or factor Xa inhibitors in patients with stable coronary, cerebrovascular or peripheral arterial disease without a clear indication for anticoagulant therapy (C6)	1 (0.8)	1 (0.8)	
Ticlopidine in any circumstances (C7)	1 (0.8)	1 (0.8)	
NSAID and vitamin K antagonist, direct thrombin inhibitor or factor Xa inhibitors in combination (C10)	2 (1.7)	0 (0.0)	
NSAID with concurrent antiplatelet agents without PPI prophylaxis (C11)	1 (0.8)	0 (0.0)	
Section D: Central nervous system			
Initiation of tricyclic antidepressants as first-line antidepressant treatment (D2)	2 (1.7)	1 (0.8)	
Selective serotonin re-uptake inhibitors with current or recent significant hyponatraemia (D4)	1 (0.8)	1 (0.8)	
Benzodiazepines for ≥ 4 weeks (D5)	15 (12.4)	6 (5.0)	
Anticholinergics or antimuscarinics in patients with delirium or dementia (D8)	3 (2.5)	2 (1.7)	

Acetylcholinesterase inhibitors with a known history of persistent bradycardia, heart block or recurrent unexplained syncope or concurrent treatment with drugs that reduce heart rate such as beta-blockers, digoxin, diltiazem, verapamil (D11)	1 (0.8)	2 (1.7)
First-generation antihistamines (D14)	2 (1.7)	0 (0.0)
Section E: Renal system		
Digoxin at a long-term dose greater than 125µg/day if eGFR < 30 ml/min/1.73m^2 (E1)	1 (0.8)	1 (0.8)
NSAIDs if eGFR < 50 ml/min/1.73m^2 (E4)	3 (2.5)	0 (0.0)
Section F: Gastrointestinal system		
PPI for uncomplicated peptic ulcer disease or erosive peptic oesophagitis at full therapeutic dosage for > 8 weeks (F2)	4 (3.3)	2 (1.7)
Drugs likely to cause constipation in patients with chronic constipation where non-consipating alternatives are appropriate (F3)	1 (0.8)	1 (0.8)
Section G: Respiratory system		
Theophylline as monotherapy for COPD (G1)	1 (0.8)	0 (0.0)
Systemic corticosteroids instead of inhaled corticosteroids for maintenance therapy in moderate-severe COPD (G2)	1 (0.8)	0 (0.0)
Section H: Musculoskeletal system		
Corticosteroids for osteoarthritis (H5)	1 (0.8)	0 (0.0)
COX-2 selective NSAIDs with concurrent cardiovascular disease (H7)	1 (0.8)	0 (0.0)
NSAID with concurrent corticosteroids without PPI prophylaxis (H8)	1 (0.8)	1 (0.8)
Oral bisphosphonates in patients with a history of upper gastrointestinal disease i.e. dysphagia, oesophagitis, gastritis, duodenitis, or peptic ulcer disease, or upper gastrointestinal bleeding (H9)	1 (0.8)	1 (0.8)
Section I: Urogenital system		
Antimuscarinic drugs for overactive bladder syndrome with concurrent dementia or chronic cognitive impairment or narrow-angle glaucoma, or chronic prostatism (I1)	3 (2.5)	2 (1.7)
Section J: Endocrine system		
Sulphonylureas with a long duration of action with type 2 diabetes mellitus (J1)	10 (8.3)	4 (3.3)
Section K: Drugs that predictably increase the risk of falls		
Benzodiazepines (K1)	52 (43.0)	24 (19.8)
Neuroleptic drugs (K2)	5 (4.1)	2 (1.7)
Vasodilator drugs with persistent postural hypotension (K3)	1 (0.8)	1 (0.8)
Hypnotic Z-drugs (K4)	9 (7.4)	4 (3.3)
Section L: Analgesic drugs		
Use of oral or transdermal strong opioids as first line therapy for mild pain (L1)	3 (2.5)	3 (2.5)

Section M: Antimuscarinic/anticholinergic drug burden			
Concomitant use of two or more drugs with antimuscarinic/anticholinergic properties (M1)	1 (0.8)	1 (0.8)	
Potential prescribing omissions[b,c]			
Any occurrence	64 (52.9)	94 (77.7)	< 0.001
Mean number (SD)	0.5 (0.5)	2.1 (1.6)	< 0.001
START criteria			
Section A: Cardiovascular system			
Vitamin K antagonists or direct thrombin inhibitors or factor Xa inhibitors in the presence of chronic atrial fibrillation (A1)	1 (0.8)	1 (0.8)	
Aspirin in the presence of chronic atrial fibrillation, where Vitamin K antagonists or direct thrombin inhibitors or factor Xa inhibitors are contraindicated (A2)	1 (0.8)	1 (0.8)	
Antiplatelet therapy with a documented history of coronary, cerebral or peripheral vascular disease (A3)	9 (7.4)	13 (10.7)	
Statin therapy with a documented history of coronary, cerebral or peripheral vascular disease, unless the patient's status is end-of-life or age is > 85 years (A5)	8 (6.6)	11 (9.1)	
ACE inhibitor with systolic heart failure and/or documented coronary artery disease (A6)	5 (4.1)	5 (4.1)	
Beta-blocker with ischaemic heart disease (A7)	4 (3.3)	8 (6.6)	
Section B: Respiratory system			
Regular inhaled β2 agonist or antimuscarinic bronchodilator for mild to moderate asthma or COPD (B1)	1 (0.8)	0 (0.0)	
Section C: Central nervous system & Ophthalamic system			
Acetylcholinesterase inhibitor for mild-moderate Alzheimer's dementia or Lewy body dementia (C3)	3 (2.5)	4 (3.3)	
Section D: Gastrointestinal system			
Fibre supplements for diverticulosis with a history of constipation (D2)	4 (3.3)	3 (2.5)	
Section E: Musculoskeletal system			
Bisphosphonates and vitamin D and calcium in patients taking long-term systemic corticosteroid therapy (E2)	9 (7.4)	9 (7.4)	
Vitamin D and calcium supplement in patients with known osteoporosis or previous fragility fractures (E3)	34 (28.1)	72 (59.5)	
Bone anti-resorptive or anabolic therapy in patients with documented osteoporosis, where no pharmacological or clinical status contraindication exists (E4)	23 (19.0)	59 (48.8)	
Vitamin D supplement in older people who are housebound or experiencing falls or with osteopenia (E5)	14 (11.6)	44 (36.4)	

Xanthine-oxidase inhibitors with a history of recurrent episodes of gout (E6)	1 (0.8)	1 (0.8)
Folic acid supplement in patients taking methotrexate (E7)	2 (1.7)	1 (0.8)
Section F: Endocrine system		
ACE inhibitor or ARB in diabetes with evidence of renal disease or microalbuminuria with or without serum biochemical renal impairment (F1)	5 (4.1)	8 (6.6)
Section G: Urogenital system		
5-alpha reductase inhibitor with symptomatic prostatism, where prostatectomy is not considered necessary (G2)	7 (5.8)	8 (6.6)

[a]Values are expressed as the number with the percentage of the total number, unless otherwise stated.

[b]Comparisons between the time at admission and discharge were performed by using Fisher's exact test and Student's t-test for categorical and continuous variables, respectively. The level of statistical significance was set at 5%.

[c]Based on the 2015 STOPP/START criteria.

ACE, angiotensin-converting-enzyme; ARB, angiotensin II receptor blocker; COPD, chronic obstructive pulmonary disease; COX, cyclooxygenase; NSAIDs, non-steroidal anti-inflammatory drugs; SD, standard deviation; SSRI, selective serotonin reuptake inhibitors; STOPP/START criteria, screening tool of older people's prescriptions and screening tool to alert to right treatment criteria.
